# Supplementary material for: A yeast phenomic model for the influence of Warburg metabolism on genetic buffering of doxorubicin
Source: Cancer Metab. 2019 Oct 23;7:9. doi: 10.1186/s40170-019-0201-3 (PMC6806529; doi:10.1186/s40170-019-0201-3)
Supplement: Supplementary file 9 — Additional file 9. GO term-specific heatmaps for REMc/GTF-enriched clusters. GO term-specific heatmaps for significant GO process terms were generated as described in methods and Figs. 3 and 4. Any related child terms are presented in subsequent pages of the parent file name. GO terms with more than 100 children, with 2 or fewer genes annotated to the term, or a file size over 300KB are not shown. All heatmaps are generated with the same layout (see Figs. 3 and 4). [file 40170_2019_201_MOESM9_ESM.bz2 › Additional_File9_GOTermHeatmaps/Additional_File9_GOTermHeatmap/pseudohyphal_growth.pdf]

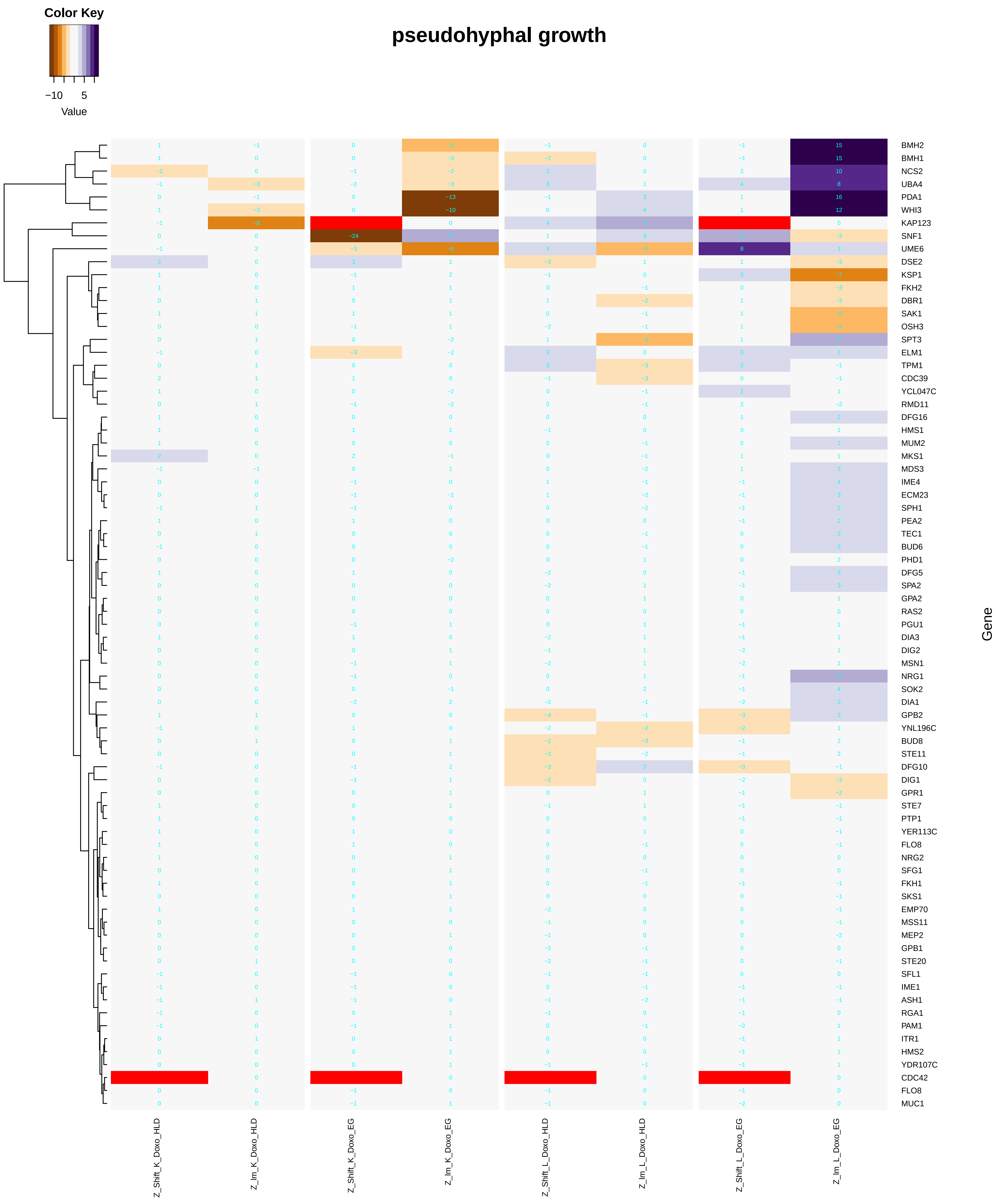

pseudohyphal growth
Color Key
−10
5
Value
Gene
BMH2
BMH1
NCS2
UBA4
PDA1
WHI3
KAP123
SNF1
UME6
DSE2
KSP1
FKH2
DBR1
SAK1
OSH3
SPT3
ELM1
TPM1
CDC39
YCL047C
RMD11
DFG16
HMS1
MUM2
MKS1
MDS3
IME4
ECM23
SPH1
PEA2
TEC1
BUD6
PHD1
DFG5
SPA2
GPA2
RAS2
PGU1
DIA3
DIG2
MSN1
NRG1
SOK2
DIA1
GPB2
YNL196C
BUD8
STE11
DFG10
DIG1
GPR1
STE7
PTP1
YER113C
FLO8
NRG2
SFG1
FKH1
SKS1
EMP70
MSS11
MEP2
GPB1
STE20
SFL1
IME1
ASH1
RGA1
PAM1
ITR1
HMS2
YDR107C
CDC42
FLO8
MUC1
Z_Shift_K_Doxo_HLD
Z_Im_K_Doxo_HLD
Z_Shift_K_Doxo_EG
Z_Im_K_Doxo_EG
Z_Shift_L_Doxo_HLD
Z_Im_L_Doxo_HLD
Z_Shift_L_Doxo_EG
Z_Im_L_Doxo_EG
